# Giant right hydronephrosis with underlying double malignancy: a case report

**DOI:** 10.11604/pamj.2023.45.21.37942

**Published:** 2023-05-05

**Authors:** Shreyas Nellamkuziyil Michael, Pirzada Faisal Masood, Umesh Sharma, Fibah Irshad Bhat

**Affiliations:** 1 Department of Urology and Renal Transplant, Atal Bihari Vajpayee Institute of Medical Sciences, Dr. Ram Manohar Lohia Hospital, New Delhi, India,; 2 Department of Pathology, Atal Bihari Vajpayee Institute of Medical Sciences, Dr. Ram Manohar Lohia Hospital, New Delhi, India

**Keywords:** Bladder tumor, giant hydronephrosis, rhabdomyosarcoma, urothelial carcinoma, case report

## Abstract

Giant hydronephrosis is mostly caused by ureteropelvic junction obstruction (UPJO). Giant hydronephrosis with concurrent malignancy is less common clinically and is easily misdiagnosed. We report a 77-year-old male who presented with sudden onset progressive abdominal distension in the last month. Abdominal computed tomography showed a right severe hydronephrotic kidney with loss of parenchymal thinning. Cystoscopy showed a 1x1cm papillary lesion protruding from the right ureteric orifice. He underwent a right radical nephroureterectomy with bladder cuff excision with lymph node dissection. Histopathology showed low-grade urothelial carcinoma of the ureter and incidental pleomorphic rhabdomyosarcoma in the right kidney. The patient refused chemotherapy and died 6 months later due to lung metastasis. Incidental pathologic finding of renal rhabdomyosarcoma in adults with giant hydronephrosis and urothelial carcinoma is a rare occurrence with diverse clinical presentations, prognoses, and outcomes.

## Introduction

Giant hydronephrosis, referred to as a hydronephrotic kidney with a content volume exceeding 1 liter, is an uncommon entity [1]. Common causes described in literature include Ureteropelvic junction obstruction (UPJO), urinary stones, trauma, renal ectopia, ureterovesical junction obstruction, and malignancies, which are rare causes [1]. Giant hydronephrosis can lead to gradual progression and comorbidities such as hypertension, rupture of affected kidneys, renal impairment, and malignant transformation in some cases [2]. Giant hydronephrosis with concurrent malignancy is less common clinically and is easily misdiagnosed [3,4]. Here, we report a case of a giant hydronephrosis of the right kidney with incidentally detected pleomorphic rhabdomyosarcoma on histopathology of the renal specimen and ureteral low-grade papillary urothelial carcinoma.

## Patient and observation

**Patient’s information:** a 77-year-old male presented with a complaint of breathlessness, which was more pronounced on lying down. It was associated with rapidly progressive abdominal distention from the last 1 month. The patient was a chronic smoker with 25 pack years of smoking. He was hypertensive and on regular antihypertensives for the last 8 years. He had a past history of transurethral resection of a bladder tumor three years back with histology of a superficial low-grade urothelial tumor without further treatment or follow-up. There was no significant family history.

**Figure 1:**
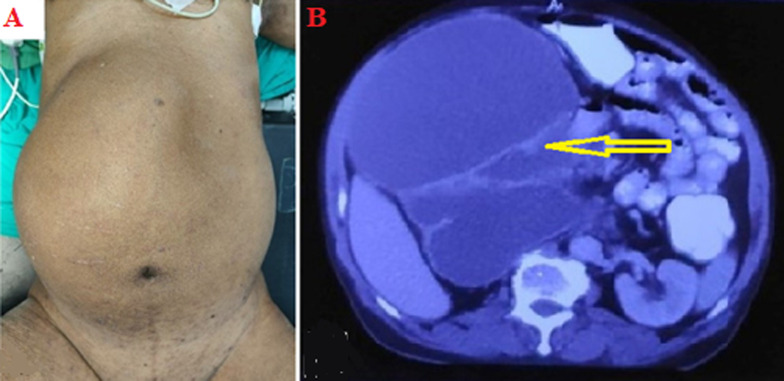
A) preoperative photo showing a distended abdomen; B) computerised tomography (CT) scan showing right giant hydronephrosis (arrow)

**Clinical findings:** on examination, the patient had a tense distended abdomen with a large abdominal mass, which was occupying the entire right hemiabdomen. The mass was non-tender and cystic in consistency with well-defined borders (Figure 1A).

**Figure 2:**
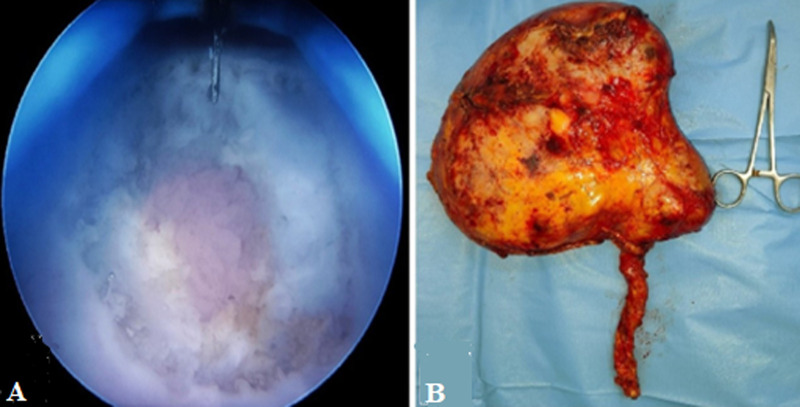
A) intraoperative photo showing transurethral bladder cuff excision; B) intraoperative photo showing a gross specimen of the resected right kidney

**Diagnostic assessment:** abdominal ultrasonography showed a 19x17x17cm homogenous septate mass in the right lumbar and umbilical region. The right kidney was not visualized separately, and the left kidney was normal. Abdominal computed tomography (CT) scan showed a right grossly hydronephrotic kidney of size 23x20x18 cm with parenchymal thinning and containing around 3.3 liters of fluid (Figure 1B). There was an abrupt cut-off in the proximal ureter with thickened enhancing ureteral wall with a maximum thickness of 2.2 cm. On cystoscopy, there was a 1x 1 cm papillary lesion seen protruding from the right ureteric orifice (Figure 2A).

**Therapeutic interventions:** with a provisional diagnosis of upper tract urothelial carcinoma with giant hydronephrosis and a non-functional kidney, the patient underwent a right radical nephroureterectomy with transurethral bladder cuff excision (Figure 2B). During surgery, extensive adhesions were seen. No abnormal vasculature was encountered. Multiple hilar, para-caval, and aortocaval lymph nodes were seen and the right template lymph node dissection was done.

**Figure 3:**
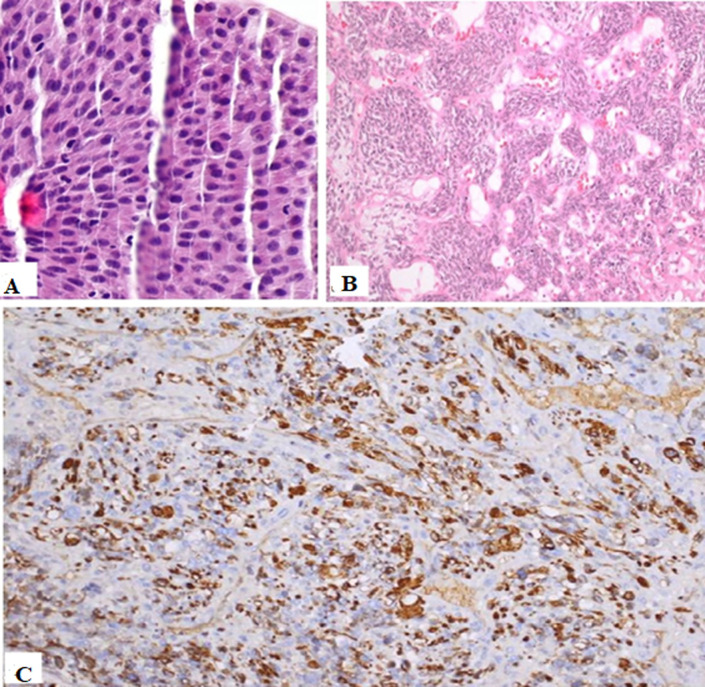
histopathologic analysis: A) low-grade urothelial carcinoma; B) spindle rhabdomyoblasts cells; C) immunohistochemistry showing desmin positivity

**The follow-up and outcomes:** the patient was discharged from the hospital on postoperative day seven. The patient developed a surgical site infection and was treated with regular dressing. The gross specimen showed a cystically dilated kidney with no normal renal parenchyma. Renal walls were thickened with multiple polypoidal nodules. The renal pelvis was filled with a tumor and the entire length of the ureter showed complete replacement by the tumor invading the entire wall thickness. Microscopy of the renal nodule showed features of pleomorphic rhabdomyosarcoma that was limited to the kidney. Light microscopy disclosed a malignant tumor made up of large, haphazardly arranged cells of various shapes with abundant, intensely eosinophilic cytoplasm. Cytoplasmic cross-striations were discovered in the spindle and tadpole-shaped cells. There were different mitotic figures and necrotic areas. The immunohistochemistry (IHC) was positive for Desmin. The histopathology of the ureter specimen showed non-invasive, low-grade papillary urothelial carcinoma (Figure 3A, B, C). Lymph nodes were negative for metastasis. The final histopathology was the concomitant presence of pleomorphic rhabdomyosarcoma of the kidney and low-grade urothelial cancer of the ureter. Chemotherapy was recommended, but the patient declined. The patient developed multiple lung metastases a month after surgery. He died 6 months later after the initial operation.

**Patient’s perspective:** during treatment, the patient and his family were satisfied with the level of care provided to him.

**Informed consent:** written informed consent was obtained from the patient’s family.

## Discussion

The giant hydronephrosis case was first published in 1746, and there are a little over 500 cases that have been published with a wide spectrum of underlying causes. UPJO has been recognized as the most common cause of giant hydronephrosis. Whereas, tumor pathology of the urinary tract is uncommon [5,6]. Despite numerous cases of massive hydronephrosis described in the literature, just a few patients presented with more than 2 liters of urine in the pelvicalyceal system. In the current case, the total amount of content was more than 3.3 liters, enough to fill the entire abdominal cavity. Regarding previous reports, Yilmaz *et al*. and Aljbri *et al*. reported cases of giant hydronephrosis having 13 litters and 12 litters of urine in the pelvicalyceal system, respectively [1,7]. Abdominal distension in the case of UPJO is usually gradually increasing but in our case, it was a short onset and progressive, raising the suspicion of some other etiology, such as malignancy [3]. Occasionally, giant hydronephrosis may present with pain, fever, respiratory distress, and even haematuria or urosepsis secondary to urine stasis and infection [1]. Suggested mechanisms for renal pelvic malignancy developments are a result of the carcinogenic effect on the mucosa caused by the long-term retention of carcinogenic urine in the renal pelvis and the stimulation of long-term chronic infection that leads to the development of carcinogenesis [3,8].

Ultrasonography may identify giant hydronephrosis as the presence of hydronephrosis which extends beyond the abdominal midline or extends to five or six vertebral [9]. Computed tomography (CT) has been the definitive diagnostic investigation in the evaluation of such cases, as performed in our patient [9]. In our patient, giant hydronephrosis may be caused by upper urinary tract transitional cell carcinoma, which is a rare scenario. There are few reports in literature wherein a ureteric mass has caused giant hydronephrosis, that too in such a short period [3,5,8]. Concomitant rhabdomyosarcoma was a pleasant surprise as adult renal rhabdomyosarcoma is a rare and aggressive entity with a paucity of data and reports in the literature and is probably the first case in the literature with giant hydronephrosis caused by urothelial carcinoma of the ureter and with a concomitant Rhabdomyosarcoma of the kidney [10,11]. On imaging study, there is no peculiar characteristic that can be ascribed to rhabdomyosarcoma. However, radiologic studies may provide useful information for surgical planning, as seen in our case [11,12].

The ideal treatment for giant hydronephrosis is usually a simple nephrectomy, because of the frequent association of foci of dysplasia, tumor changes in the parenchyma, and collecting system as a result of chronic irritation [1]. In our case, we performed radical nephroureterectomy with bladder cuff excision because of high-risk upper tract urothelial cancer. Rhabdomyosarcoma is divided into 4 histopathologic subtypes: embryonal, spindle cell/sclerosing, alveolar, and pleomorphic types. Pleomorphic rhabdomyosarcoma has been the most commonly encountered rhabdomyosarcoma in adult population as per the literature. This subtype of tumor cells expresses desmin, CD56, myogenin, and vimentin, but not PAN-CK, LCA, S100, CD34, inhibin, EMA, and SMA. In our case, the pleomorphic rhabdomyosarcoma expressed Desmin positivity, which is the commonest IHC marker positivity seen in any RMS. Other IHCs done were PanCK and SMA, which came out to be negative [13]. After nephrectomy, adjuvant chemotherapy with vincristine, dactinomycin, and cyclophosphamide should be started for rhabdomyosarcoma cases. Radiation therapy may be utilized for residual tumors and localized recurrences [11,12]. However, our patient refused any further treatment and died 6 months later due to lung metastasis. The rapid development of lung metastases in this case, despite complete resection of the primary tumor, suggests that these patients may be at a higher risk of poor outcomes and would benefit from early aggressive multimodal therapy, a similar case of rapid metastasis was mentioned by Lin *et al*. [11].

## Conclusion

Incidental pathologic finding of renal rhabdomyosarcoma in adults with giant hydronephrosis and urothelial carcinoma is a rare occurrence with diverse clinical presentations, prognoses, and outcomes.
